# "Quality of life of patients after surgical treatment of cervical spine metastases"

**DOI:** 10.1186/s12891-016-1175-8

**Published:** 2016-07-26

**Authors:** Grzegorz Guzik

**Affiliations:** Department of Orthopaedic Oncology, Specialist Hospital in Brzozów- Podkarpacie Oncology Centre, Bielawskiego 18, 36-200 Brzozów, Poland

**Keywords:** Metastases, Spinal tumours, Spinal surgery, Resections of spinal tumours, Spine stabilizations, Cervical spine

## Abstract

**Background:**

Metastases of malignant neoplasms to the cervical spine are relatively rare. The most common symptom of metastatic disease is pain. Symptoms associated with roots damage or spinal cord compression indicate locally advanced disease. In a large number of patients, surgical treatment brings benefits such as pain reduction and improvement of the quality of life. Pain intensity, neurological status, and quality of patients’ lives are measured with the VAS, Frankel, and Karnofsky scales.

**Methods:**

Symptoms of the disease, morphology of the metastasis and treatment outcomes were evaluated in 57 patients treated surgically because of metastases to the cervical spine over the period 2010–2014 in Brzozów. The morphology of the metastases was assessed on the basis of CT and MR examinations. Pre- and postoperative functional status of the patients was evaluated using Karnofsky scale. The intensity of pain was assessed with VAS and the neurological status was evaluated by using Frankel’s grades. Anterior approach was employed in 16 patients, posterior approach in 30 patients, and postero-anterior approach in 11 patients. The inter-group differences were evaluated using the U Mann–Whitney and Wilcoxon Matched Pairs test. All statistical analyses were performed by using Statistica 10. A value of *P* < 0.05 was considered statistically significant.

**Results:**

The majority of patients suffered from pain associated with instability of the spine. Multi-level metastases were noted in 40 patients, while in 17 patients 1 vertebra was involved. In 51 patients the metastases caused pathological fractures of the vertebrae. The most common neurological complications was observed in patients with multi-level tumors and with pedicles involvement. After surgery patients functional status improved and pain intensity decreased. The best results (statistically significant) were observed in patients operated with anterolateral approach. Complications were scarce. Two patients required reoperation due to infection.

**Conclusions:**

Surgical treatment of metastases to the cervical spine gives good outcomes and it ought to be a treatment of choice. Proper and multifaceted qualification of the patients for different treatments is of vital importance.

## Background

The most common tumors involving the spine are breast, prostate, kidney, lung cancer, and myeloma. The majority of tumors are found in the thoracic and lumbar spine, followed by the cervical spine, accounting for 17 % of cases [1–4].

There are various spinal cord compression symptoms, of which severe spinal pain is most significant. Postural pain resulting from spinal instability is frequently reported. The symptoms are more pronounced in the mobile sections of spine, such as the cervical and lumbar as well as the cervicothoracic and thoracolumbar junctions. Neurologic deficits of various severity are noted in 60 % of patients. Pareses are observed in approximately 30–50 % of patients, most commonly when the lesions involve the thoracic spine [1–4].

Diagnostics is based on careful clinical investigation of the patient and should be complemented by radiographic examination. In most cases, an accurate of the extent of malignancies and the risk of complications requires performing CT and MRI [1, 2, 6–8].

Qualification for the treatment need comprehensive evaluation of the general condition, stage of the neoplastic disease and prognosis [1, 2, 9].

Treatment goals should be: improvement in the quality of life; pain relief and reducing the risk of neurological complications. To assess pain intensity the researchers use many different scales, but most commonly - VAS score. The Frankel scale adequately determines severity of neurological deficits. The Karnofsky scale is a valuable tool to perform measurement of the quality of life [10–17].

The most common treatment method is radiotherapy. Local tumor control and pain relief have been achiev in most cases. Radiotherapy is ineffective in patients with vertebral fractures, spinal instability or neurologic deficits [1, 2, 9].

Surgeries of spinal metastases are increasingly widely performed and their results are encouraging. Resections of metastatic tumours significantly reduce the risk of local recurrences of tumor and are reserved for patients in good general condition and with long-term prognosis. Bone losses are filled with prostheses or bone cement. Usually, spine stabilizations are multisegmental and aim at quick recovery and improving walking status [2, 8, 18–21].

## Methods

Between 2010 and 2014 at the Department of Orthopaedic Oncology in Brzozów, a total of 542 patients with spinal tumours were treated, of whom 474 underwent surgeries. 57 patients received surgery treatment for the metastases localized in the cervical spine.

Records of orthopaedic, neurologic and imaging examinations were analyzed. Before the surgery, standard radiograms, CT scan of the spine and MRI were performed in all patients. The following clinical factors were assessed: the type, localization and extension of pathological lesions, their relation to the adjacent structures, especially spinal cord and nerve roots. The quality of bone tissue, spinal axis disorders, type of the fractures, stability of spinal segments or their dislocations were considered. After analysis of metastasis morphology the optimal treatment strategy including surgical approach, extension of tumour resection, and spine fixation was selected.

Preoperative and postoperative intensity of pain was assessed using visual analog pain score scale (VAS). The ability to function independently and the functional status were evaluated using Karnofsky scale. The assessment of the ability to move and neurological status were carried out using the Frankel grading.

Posterior approach was employed in 30 patients for spine stabilization and indirect decompression. Lateral mass screws were placed 2 levels above and below the pathological level. Subsequently in 29 patients wide laminectomy was performed. Craniocervical stabilization was performed in 18 patients, and 12 other received cervical or cervicothoracic stabilization.

In 16 patients the surgery was performed through left sided anterior approach, described by Southwick and Sundaresan. Anterior approach was performed in patients with a single metastasis. After the tumour and vertebral body resection, bones were reconstructed with titanium prostheses (in 6 patients), PEEK (in 4 patients) or PMMA (in 6 patients).

A total of 11 patients were operated on using two approaches at the same time. The indication for combined approach was massive tumours involving two (9 patients) or three vertebrae (2 patients).

On postoperative day 14, the condition of the patients was assessed taking into account VAS pain intensity, Karnofsky functional status and neurological status evaluated in accordance with Frankel grading system. The pre- and postoperative examination results were compared.

The inter-group differences were evaluated using the U Mann–Whitney test. Results of intervention were compared using the Wilcoxon Matched Pairs test. The categorical variables were expressed as percentages. The inter-group differences were tested using the *χ*2 test. All statistical analyses were performed by using Statistica 10. A value of *P* < 0.05 was considered statistically significant.

## Results

The majority of the treated patients were women, representing 64 % of patients. The mean age was 62 years for women (range from 38 to 84 years), and 66 years for men (range from 44 to 87 years).

In 4 patients, spinal tumour was the first sign of neoplastic disease and orthopaedic treatment helped establish histopathological diagnosis. Breast cancer was most common malignancy −31 cases, followed by multiple myeloma- 9 cases, lung cancer- 6 cases, kidney cancer- 5 cases, thyroid cancer- 2 cases.

In 40 patients, the lesions involved two or more vertebrae. Metastatic lesions localized in one vertebra were noted in 17 patients. The metastases involved both posterior and anterior elements of the vertebrae occurred in 42 patients, while in 2 patients only posterior elements were involved – Table 1. Pathological fractures were diagnosed in 51 patients. In 6 cases metastatic lesions caused no fractures. Clinical and radiological instability were observed in 51 patients. Imaging examinations revealed spinal canal stenosis in 35 patients and in 6 of them infiltrate the dura mater.

**Table 1:** The table shows the morphology of the metastasis in correlation with the treatment method applied

Morphology	Anterior stabilization (A)	Posterior stabilization (P)	Stabilization through both approaches (H)
1 vertebra	16	1	-
2 or more vertebrae	-	29	11
Vertebral body	13	-	-
Posterior elements	-	2	-
Posterior and anterior elements	3	28	11

Neurogenic pain was observed in 27 patients. Somatic night pain affected 23 patients. The predominant symptom was pain associated with the instability of the spine in 39 patients. Often, various types of pain coexisted. The preoperative mean pain intensity score in VAS scale was 7.2 (range: 3–10).

The mean Karnofsky performance score, reflecting the patients’ functional status and their independence, was 54.5 (range: 30–100).

The preoperative neurological examination found neurological deficits in 27 patients. Complete paralysis of limbs were diagnosed in 3 patients (Frankel A). Major pareses were detected in 11 patients and they was classified as Frankel grade B, while 10 patients were classified as Frankel C grade. Minor pareses (Frankel grade D) occurred in 3 patients - Table 2.

**Table 2:** The table shows the morphology of the metastatic lesions in the cervical spine in correlation with the neurological status and spinal canal stenosis signs seen on MRI scan

Morphology	Frankel A	Frankel B	Frankel C	Frankel D	Frankel E	Stenosis
1 vertebra	-	-	1	2	14	3
2 or more vertebrae	3	11	9	1	16	32
Vertebral body	-	1	-	2	10	3
Posterior elements	-	-	1	1	-	2
Posterior and anterior elements	3	10	7	-	20	30

The pain, rated on a VAS scale, decreased after the surgical treatment. The mean score of pain intensity level was 4.3. On postoperative day 14, the mean Karnofsky performance score was 64.7 points. In a group of 14 patients with paralyses or deep pareses, unable to walk before the surgery, 4 patients restored an upright position and they started walking on crutches or with a frame. A total of 13 patients showed neurologically improved – Table 3.

**Table 3:** The table shows the parameters evaluated in patients in conjunction with the method of surgical treatment

	Anterior stabilization (A)	Posterior stabilization (P)	Anterior and posterior stabilization (H)
Neurological function improvement	2	4	7
VAS score before the surgery	5.2	7.9	8.5
VAS score after the surgery	2.1	4.6	6.1
Karnofsky score before the surgery	70.1	47.8	45.7
Karnofsky score after the surgery	80.3	58.2	55.6

Table 4 presents a statistical analysis of pain intensity and functional status before and after surgical treatments. There were significant statistical differences in the severity of pain and efficiency between patients group P (posterior fixation), and group A and H (front stabilization and hybrid stabilization).

**Table 4:** Statistical analysis of the VAS and Karnofsky results before and after the surgical treatment

Approach	VAS before the surgery	VAS after the surgery	*P* value	Karnof’sky before the surgery	Karnof’sky after the surgery	*P* value
(A) Anterior *N* = 16	5.2 ± 1.7	2.1 ± 1.1	<0.05	70 ± 14	80 ± 12	<0.05
(P) Posterior *N* = 30	7.9 ± 1.4*	4.6 ± 1.2*	<0.05	48 ± 11*	58 ± 10*	<0.05
(H) Hybrid *N* = 11	8.5 ± 1.8	6.1 ± 1.5	<0.05	46 ± 18	56 ± 17	NS

Results are presented as a mean ± standard deviation. *p < 0.05 for inter-group difference A vs. B or A vs. C

The incidence of deep pareses (Frankel A, B) and spinal cord compression were significantly higher in patients with multilevel and pedicle involvement - Table 5.

**Table 5:** Statistical analysis of deep pareses and spinal cord compressions

Group	Percent of patients	Frankle D and E	Stenosis
One level involvement	30 %	94 %	18 %
Multi level involvement	70 %	43 %*	80 %*
Vertebral body involvement	24 %	92 %	23 %
Vertebral body and pedicles involvement	72 %	50 %*	75 %*
Pedicles involvement	4 %	50 %	100 %

Results are presented as a percent. *p < 0.05 for inter-group difference Chi X^2^

In two patients tumour infiltrate into the dura mater, and a leak of cerebrospinal fluid occurred during laminectomy and require suturing of dura mater and application of TachoSil. No clinical signs of cerebrospinal fluid leak were noted after the surgery. No intraoperative damage of the neck organs, vessels, nerves roots or neurological decline were observed. Surgical wounds were affected by minor infections in 3 cases, healed with no need for revision. Surgical revision was required in 2 cases due to pus leaking.

Four patients after posterior stabilization of the spine and 8 patients after cervicocranial stabilization reported chronic headaches. Amongst two patients after cervicocranial stabilization transient dizziness and dysequilibrium were observed, however, regression of these symptoms was noted after 2–6 days.

Increased muscle tone of paraspinal and neck muscles persisted in all patients after cervicocranial stabilization. They also reported difficulties with daily tasks such as washing, combing, writing and reading resulting from a forced head position – Table 6.

**Table 6:** The type and the incidence of complications after different surgical treatments

	Anterior stabilization (A)	Posterior stabilization (P)	Anterior and posterior stabilization (H)
Damage to the dura mater	-	2	-
Headache	-	12	-
Dizziness	-	2	-
Increase of muscle tone	-	18	-
Daily task difficulties	-	18	-
Surgical wound infections	-	4	1

The patients who had undergone surgeries through anterior approach improved their functioning status faster than other groups. Moreover their postoperative pain was reduced, and mobility of the spine was limited to a various degree, but they could perform the basic activities of daily living.

The patients with a single metastasis were qualified for surgeries through anterior approach. In this group no neurological impairments occurred including paralyses and deep pareses, which predetermined better treatment outcomes.

The patients with locally most advanced stage of the disease need combine approaches to perform adequate surgical decompression. The majority of them had significant neurological deficits and comparatively slightest improvements in their quality of life.

No major structural failures, rods or prostheses dislocations, nor fractures of the rods were found in patients during follow-up.

## Discussion

Spinal metastases usually occur in elderly people in their sixth or seventh decade of life. The improving detectability and effectiveness of treatment of malignant neoplasms result in a prolonged survival of patients, but the numbers of bone metastases arise [1, 2].

The most important outcome of surgical treatment of spine metastases is improvement quality of live. Pain relief enables patients to return to an active life. Restored spinal stability allows patients to assume an erect position without orthopedic corsets. The incidence of deep neurological complications is significantly lower. The majority of patients are able to walk and to live independently. They rarely need treatment in health care units [2, 22, 23].

It is now believed that radiotherapy as the sole treatment method should be reserved for patients with severe conditions who are disqualified from surgical treatment [2, 4, 24].

Resections of vertebral bodies together with tumours are usually performed in patients with single metastasis whose general condition and prognosis are good. Surgeries through posterior or combined approach are performed in patients with large tumours causing multiple fractures, spinal instability, and nervous structures compression [2, 4, 5, 19, 25–27].

Bauer’s study has found that the quality of life, prognosis and survival improve in patients after surgical treatment of metastases to the spine [20].

According to Guzik, a combination of surgical treatment and postoperative radiotherapy opens up the possibility of local control of the tumour and prevents local recurrence [2].

Weigel and Maghsudi point out effectiveness of surgical treatment of the spine tumours in patients in good general condition. Mainly in pain reduction and the motor function improvement [8].

An objective assessment of the quality of patients’ lives is a very serious challenge. The factors that impact mostly on QoL are: pain; patients’ fitness; ability to move unaided and to perform activities of daily living independently; mental condition [2, 4, 6, 7, 15, 20].

Our study evaluated the pre- and postoperative intensity of pain, functional and neurological status of the patients in the context of the impact on the quality of life.

As a subjective experience, pain is particularly difficult to assess. The best method for evaluating and comparing the intensity of pain is to use different scales (VAS, NRS, MPAC, and others). It is important that the scales are manageable and easy to understand for patients. The VAS scale was used in our study. This scale is more sensitive than verbal pain intensity scale and allows to measure pain of low intensity [10–13].

Patients’ functional performance was evaluated according to the Karnofsky scale. The Frankel scale was used for neurologic assessment. The Karnofsky scale helps to make initial decisions concerning qualification for the treatment. The author of this scale proposed the following criteria: radical surgeries in patients with scores of more than 70 points; palliative surgery (decompression of the nervous structures and spine fixation) is recommended for patients with scores between 70 and 40 and non-surgical palliative treatment should concern patients with a score below 40 [14, 15, 17].

Postoperative medical care must involve orthopaedic management and early rehabilitation. Radiotherapy is recommended 3 weeks after the surgery, when wound is completely healed [1, 2]. Postoperative complications were scarce.

In our study the functional and neurological status of the patients improved after the surgery. Consequently, the quality of patients’ lives improved. This proves the effectiveness of the surgical treatment. The best results were obtained in patients undergoing anterior and hybrid approach. The incidence of neurological deep deficits was higher in patients with multi-level tumors and with pedicle involvement.

## Conclusions

1. Surgical treatment of metastases to the cervical spine is one of the therapeutic options which should by considered.
2. Surgical resection of the metastasis and stabilization of the spine result in decreased pain intensity, improved quality of life, and in some cases, reduced neurological deficits.
3. Qualification for the surgery is multifaceted and should be individual for each patient.

## Abbreviations

MPAC, memorial pain assessment card; NRS, numeric rating scale; PEEK, polyether ether ketone; PMMA, polymethyl methacrylate; VAS, visual analogue scale
